# Correction: Information technology capability, open technological innovation and firm growth

**DOI:** 10.1371/journal.pone.0296601

**Published:** 2024-01-02

**Authors:** The PLOS One Staff

There are errors in the Funding section. The correct Funding statement is: This work was supported by the National Natural Science Foundation of China, Research on the Intelligent Mode and Path of Manufacturing Industry Integrating into the Digital Economy (Grant 72372121) awarded to LL. The funder had no role in study design, data collection and analysis, decision to publish, or preparation of the manuscript.

The publisher apologizes for the error.
