# Supplementary material for: Information technology capability, open technological innovation and firm growth
Source: PLoS One. 2023 Oct 24;18(10):e0291227. doi: 10.1371/journal.pone.0291227 (PMC10597510; doi:10.1371/journal.pone.0291227)
Supplement: Attachment — Submitted filename: PONE-D-22-14268_reviewer.docx [file pone.0291227.s002.docx]

**PLOS ONE**

**Information Technology Capability, Open Technological Innovation and Firm Growth**

**Manuscript Number: PONE-D-22-14268**

**Introduction and literature review**

Indeed, the introduction to this article is very well written, but I have a few recommendations for you that must be implemented.

i. The introduction section must offer the study gap and objectives of the study.
ii. Highlight current work contributions.
iii. The selection of variables for the current study must be elaborated briefly.
iv. Recent evidence should be presented to support the study's hypotheses.
v. “On the other hand, novelty is essential for overcoming resource constraints and establishing a competitive advantage (Anwar et al., 2018)” Pay attention to citations; it is not necessary to bring up unrelated facts.
vi. Relevant references, including introduction, literature review, and hypothesis development, must be from within the last five years, except for theories and books.

**Conceptual model**

i. I would recommend that you create a table before the research framework and mention previous studies from the last five years, including methodology, variables, and countries, and then draw your research gap.

**Methodology**

i. In the field of social studies, is a sample size of 28.7 percent acceptable? strengthen your argument with references to pertinent sources.
ii. Sample size calculation must be performed and reported with the use of Gpower 3.1 software.

**Variables and measures**

Create a table for variables and mentions, variable names, number of items, and previous authors.

| Variables | No. of items | Author's (previous authors) |
| --- | --- | --- |

**Discussion and Conclusion**

i. Compare the results of your study with previous studies in the discussion and conclusion section.
ii. Contributions and limitation & future research should be inline.
