# Supplementary material for: Information technology capability, open technological innovation and firm growth
Source: PLoS One. 2023 Oct 24;18(10):e0291227. doi: 10.1371/journal.pone.0291227 (PMC10597510; doi:10.1371/journal.pone.0291227)
Supplement: Attachment — Submitted filename: Response to Reviewers.docx [file pone.0291227.s005.docx]

Response to Reviewers

**Dear Editor and Reviewers:**

Thank you for your letter and the reviewers’ comments on our manuscript titled “Information Technology Capability, Open Technological Innovation and Firm Growth” (ID: PONE-D-22-14268R1). We appreciate the valuable and constructive comments, which have guided us to improve and refine our paper. We have meticulously reviewed the suggestions and incorporated the necessary changes, which are highlighted throughout the revised paper. The minor revisions made in the manuscript in response to the reviewers’ comments are outlined below:

**Responds to the reviewers’ comments:**

**Reviewer #1**

No Response

**Reviewer #2**

Thank you for submitting your article on the effects of Information Technology capability on firm growth in the context of open technological innovation. The findings are interesting and contribute to the literature on IT capability and firm growth. The research is also sound and has possible policy implications. All previous comments have been adequately addressed but it needs some minor revisions, which are recommended as follows:

**Comments:**

1.	The methodology section is good; however, the authors should justify the use of regression analysis for the estimations. The study is based on a cross-sectional design which limits the ability to draw causal conclusions as mentioned by the authors under the limitations of the study.

Reply: Thank you for your valuable feedback on our manuscript. We appreciate your positive remarks regarding the methodology section. We understand your concern regarding the use of regression analysis in our study, especially considering its cross-sectional design. We would like to address this concern and provide further justification for our choice of analysis.

While we acknowledge that cross-sectional designs have inherent limitations in establishing causality, they are commonly used in many social science and survey research studies. In our case, a cross-sectional design was appropriate as it allowed us to capture a snapshot of the variables of interest at a specific point in time. Our primary goal was to examine the associations and relationships between these variables, rather than establish causal links.

Regression analysis, despite its limitations in determining causality, remains a widely accepted statistical method for exploring relationships between variables. By utilizing regression analysis, we were able to assess the strength and direction of these relationships, controlling for potential confounding factors. This analytical approach allowed us to generate meaningful insights into the associations observed in our data.

To ensure transparency, we have included a discussion in the limitations section of our manuscript, acknowledging the limitations of the cross-sectional design and the potential for reverse causality or unmeasured confounders. We emphasize the need for further longitudinal research to establish causal relationships and address these limitations.

We hope that our explanation clarifies the rationale behind our use of regression analysis in a cross-sectional design. We appreciate your feedback and are open to any further suggestions or recommendations you may have to strengthen our manuscript.

(**Please see** the edit texts on page 10 in Section 3.4 Data analysis methods)

2.	Please check Line 390 under “Summary statistics and correlations”, the Table number needs to be changed (Table IV instead of Table II). If possible, the authors may divide Table IV into two separate tables.

Reply: Thank you for bringing this to our attention and providing valuable feedback on our manuscript. We apologize for the oversight in the labeling of the table. We have made the correction and ensure that the correct table number (Table IV) is reflected in the revised version of the manuscript.

(**Please see** the edit texts on page 12 in Section 4.2)

Regarding your suggestion to divide Table IV into two separate tables, we appreciate your recommendation. However, we have decided to maintain the current table formatting and present the data as a combined table. By doing so, we believe we can still present the information in an organized and concise manner, allowing readers to navigate and comprehend the data effectively. We have carefully considered your suggestion, but ultimately determined that preserving the existing table format would be more efficient for conveying the findings.

3.	The generalizability of the findings to other contexts may be limited due to a relatively smaller sample size. The Authors can further justify it or present it in the limitation section.

Reply: Thank you for your insightful comments and feedback on our manuscript. We appreciate your concern regarding the generalizability of our findings due to the relatively smaller sample size. We agree that addressing the limitations of our study, including sample size, is crucial for providing a comprehensive understanding of the scope and applicability of our findings.

In the revised version of our manuscript, we will explicitly address the limitations associated with the sample size in the limitation section. We will discuss the potential impact of the smaller sample size on the generalizability of our findings and acknowledge that caution should be exercised when extrapolating our results to broader populations or different contexts.

While our study sample was carefully selected and represented a specific population of interest, we acknowledge that a larger and more diverse sample would strengthen the external validity of our findings. We will emphasize the need for future research to replicate our study using larger sample sizes and encompassing a wider range of participants, settings, or contexts to enhance the generalizability of the results.

We appreciate your valuable input in helping us improve the clarity and completeness of our manuscript.

(**Please see** the edit texts on page 17 in Section 5.4 Limitations and future research)
