# Supplementary material for: Information technology capability, open technological innovation and firm growth
Source: PLoS One. 2023 Oct 24;18(10):e0291227. doi: 10.1371/journal.pone.0291227 (PMC10597510; doi:10.1371/journal.pone.0291227)
Supplement: Attachment — Submitted filename: Review.docx [file pone.0291227.s006.docx]

**Manuscript Title: Information Technology Capability, Open Technological Innovation and Firm Growth**

**Manuscript Number: PONE-D-22-14268R2**

**Comments to editor:**

Dear Editor,

I hope this message finds you well. I am writing to express my strong recommendation for the acceptance of the manuscript titled "Information Technology Capability, Open Technological Innovation, and Firm Growth" for publication in the Journal. Having carefully reviewed the manuscript, I am impressed by the rigorous research approach and its contributions to the intersection of information technology capability, open technological innovation, and firm growth. The study's focus on these crucial areas of business and technology aligns well with the journal's scope and the interests of its readership.

The manuscript demonstrates a robust research methodology and an in-depth analysis that contributes significantly to understanding how information technology capabilities and open technological innovation strategies impact firm growth. The results and findings presented in the manuscript offer practical implications for both academics and practitioners, emphasizing the importance of strategic IT investments and innovative approaches for fostering firm growth in a competitive landscape. I am confident this manuscript will be a valuable addition to the journal, advancing the field's knowledge and serving as a reference for future research endeavors. Therefore, I strongly recommend accepting the manuscript for publication.

Thank you for considering my recommendation. I look forward to seeing this important research reach a wider audience through the pages of your esteemed journal.

Best regards,

Dr. Muhammad Imran

Faculty of Technology Management & Business,

Universiti Tun Hussein Onn Malaysia
