# Supplementary material for: Information technology capability, open technological innovation and firm growth
Source: PLoS One. 2023 Oct 24;18(10):e0291227. doi: 10.1371/journal.pone.0291227 (PMC10597510; doi:10.1371/journal.pone.0291227)
Supplement: Attachment — Submitted filename: Referee Report Plos One.docx [file pone.0291227.s003.docx]

**Title: Information Technology Capability, Open Technological Innovation and Firm Growth**

**Manuscript Number: PONE-D-22-14268**

This study investigates the influence of open technological innovation, one of the potential mediators, in the link between IT capability and firm growth in China by using cross-sectional data of 259 ventures. The study finds that IT capability, including flexibility and integration of information technology, has a significant factor in firm advancement. Also, results confirm that open technological innovation plays a significant mediating role between IT integration and firm growth. The study recommended that IT integration needs the Internet-information-system can be flawlessly combined with the system of innovation partners, and can easily access, exchange and aggregate the resources and data of innovation partners, so as to integrate and absorb.

Although the paper does follow a regular type of analysis for paper implementing theoretical/empirical mechanisms studies, I was not really impressed by the total picture of it. After all, the author(s) themselves recognize that their novelty comes from the fact that the theoretical mechanisms have not dealt with this country sample. I am afraid though that the paper in its current form is way too underdeveloped both theoretically and empirically. It seems that the author(s)would greatly benefit by submitting their work to some conferences to get feedback and to their colleagues for some reviews. There could be merit in what you are trying to explain, to my understanding open technological innovation and how/when it affects positively or not on firm growth and capability. Even then, the paper suffers from a number of weaknesses that must be explicitly considered in improving the picture of it. In particular:

1. The introduction includes one and half page that do not communicate well with each other. The introduction is mostly focused on general background, while it should be specific with target firms and country. The work is not well written and shows flaws in the structuring of logical arguments without highlighting the original pizzle. Authors need to make clearer what we have learned from previous studies and what we are uncertain about. There are many more! this research needs to build on these and demonstrates very clearly how you build on and extend this stream of research. The second research question is also not new. For example, the study indicated that using IT capacity instead of IT investment capacity is significant contribution however this couldn’t unique contribution. Because investment and capacity can be used robust analysis to allow you to make better decisions. Also, there are studies who investigate the relationship between IT capacity and firm performance, e.g., Liu et al. (2013, Felipe et al. 2019 etc.). It is widely accepted that the relationship between IT innovation and firm growth and there are numerous studies on this. In what ways do your conceptualizations advance this literature? Also, the target country is China, how this study could impact globally. Authors should explain what the importance of this study is. I mean the paper should incorporate clearer motivation and background of the issue and its importance.
2. There is no theoretical model or offers no innovation since it has been well established in the relevant literature. Your argumentation of the hypotheses is not sufficient. Your need to develop a theoretical framework and follow the same line to develop all the three arguments. At the moment, there is really nothing useful there. It is not convincing at all. Also, your hypotheses refer to Chinese firms - there is no generalization there. e.
3. It would be helpful if you could relate your hypotheses in a theoretical framework and follow this up with a clearly pronounced hypotheses that relates to the theoretical argument made prior. The operationalisation of the hypothesis is not part and parcel of why you argue this particular hypothesis and should be presented in the methods section. The selection and justifications of the variables should be further discussed with supporting references.
4. The study used the survey data of 256 new ventures out of 2854 valid surveys. However, there should discussion about the validity of the surveys. Also, why chosen provinces were Beijing, Tianjin, Shandong Jiangsu and Hebei?
5. In addition, the model could be used to identify the relationship between the variables with existing literature. For example, the section 3.2 should be linked with hypotheses and expected connection between the variables. Although, authors mentioned that they followed previous studies, Rai, 2010; Cui, 2015; Bi et al., 2017, for variables selection.
6. The paper at its current version is a-theoretical. There is not a single theory you are basing your arguments or attempting to develop and inform through your research. Your paper could be drawing from International Business/finance theory (innovation) and learning from firm growth. It needs to draw on particular mechanisms and explain how/when/why growth in the domestic context is enhances when firms use IT capacity.
7. Empirical findings supports the hypotheses however, there should be critical analysis and supported with existing literature. Therefore, the author is suggested to reconstruct the theoretical and empirical model to become more realistic.
8. Conclusion should not repetition of the results. Policy implications are not very clear.
9. The above points are fundamental and need to be addressed very carefully. I would strongly encourage the author(s) to take ample time to rethink their study, read more papers from good and reputed journals and try to completely re-write their study. I wish you all the best of luck.

Reference

Liu, H., Ke, W., Wei, K.K. and Hua, Z. (2013). The impact of IT capabilities on firm performance: The mediating roles of absorptive capacity and supply chain agility. Decision Support Systems, 54(3), pp. 1452-1462.

Felipe, C.M., Leidner. D.E., Roldán, J.L., Leal-Rodríguez, A.L. (2019). Impact of IS Capabilities on Firm Performance: The Roles of Organizational Agility and Industry Technology Intensity.
